# Supplementary material for: Efficacy of Platelet-Rich Plasma in Women with a History of Embryo Transfer Failure: A Systematic Review and Meta-Analysis with Trial Sequential Analysis
Source: Bioengineering (Basel). 2023 Feb 27;10(3):303. doi: 10.3390/bioengineering10030303 (PMC10045363; doi:10.3390/bioengineering10030303)
Supplement: Supplementary file 1 [file bioengineering-10-00303-s001.zip › bioengineering-2146574-supplementary.pdf]

bioengineering

MDPI

Table S1. Outcome definitions and time of measurement.

Study	Time of Measurement	Definition
Allahveisi et al., 2020	Implantation rate	dividing the number of the observed embryonic sacs in the 6-week-old sonogram by the number of the transferred embryos.
	Biochemical pregnancy	NA
	Clinical pregnancy	The rate of clinical pregnancy was recorded by dividing the number of fetal poles with an observed heartbeat in the 6-week-old sonogram by the number of the transferred embryos
	Live birth rate	24 weeks of gestation was considered as live birth
	Miscarriage rate	NA
Eftekhar et al., 2018	Implantation rate	The ratio of gestational sacs to the number of embryos transferred
	Biochemical pregnancy	The chemical pregnancy was defined as serum B-hCG ≥50 IU/L after 14 days from embryo transfer.
	Clinical pregnancy	The clinical pregnancy as the presence of a gestational sac with heartbeat identified by ultrasound 5 weeks after the embryo transfer
	Live birth rate	pregnancy as pregnancy continued after 20 weeks
	Miscarriage rate	The abortion rate as clinically recognized pregnancy losses before 20 weeks of gestation
Ershadi et al., 2022	Implantation rate	NA
	Biochemical pregnancy	Chemical pregnancy was detected using positive serum β-HCG levels 2 weeks after the embryo transfer.
	Clinical pregnancy	Clinical pregnancy was detected by the presence of a heart rate on transvaginal ultrasound 5 weeks after the embryo transfer
	Live birth rate	NA
	Miscarriage rate	NA
Nazari et al., 2020	Implantation rate	NA
	Biochemical pregnancy	Chemical pregnancy and clinical pregnancy were determined by positive serum b-HCG, 2 weeks after embryo transfer
	Clinical pregnancy	The presence of fetal heartbeat in transvaginal ultrasound 5 weeks after embryo transfer.
	Live birth rate	Definition not available
	Miscarriage rate	NA
Nazari et al., 2019	Implantation rate	NA
	Biochemical pregnancy	serum βHCG 2 weeks after embryo transfer
	Clinical pregnancy	The presence of fetal heartbeat in transvaginal ultrasound 5 weeks after embryo transfer
	Live birth rate	NA
	Miscarriage rate	NA
Nazari et al., 2021	Implantation rate	NA
	Biochemical pregnancy	positive serum β-hCG after 2 weeks from the day of embryo transfer
	Clinical pregnancy	the presence of fetal heartbeat in transvaginal ultrasound after 6 weeks from the day of ET
	Live birth rate	Definition not available

	Miscarriage rate	NA
Nazari et al., 2022	Implantation rate	NA
	Biochemical pregnancy	NA
	Clinical pregnancy	Clinical pregnancy was defined by the presence of an embryonic sac at 5-6 weeks gestation.
	Live birth rate	Live birth was defined as birth after 24 weeks of gestation
	Miscarriage rate	Spontaneous abortion was defined by the loss of pregnancy before 20 weeks of gestation
Obidniak et al., 2017	Implantation rate	Definition not available
	Biochemical pregnancy	Definition not available
	Clinical pregnancy	Definition not available
	Live birth rate	Definition not available
	Miscarriage rate	Not definition available
Zamaniyan et al., 2020	Implantation rate	Number of gestational sacs on ultrasound per the total number of embryos transferred into the uterine cavity
	Biochemical pregnancy	Serum b-HCG two weeks after frozen-thawed embryo transfer
	Clinical pregnancy	The presence of fetal heartbeat in transvaginal ultrasonography five weeks after frozen-thawed embryo transfer
	Live birth rate	NA
	Miscarriage rate	Definition not available
Zargar et al., 2021	Implantation rate	Definition not available
	Biochemical pregnancy	NA
	Clinical pregnancy	Definition not available
	Live birth rate	The delivery of a live born child after 24 weeks of gestational age
	Miscarriage rate	Definition not available

NA: Not applied.

Table S2. Excluded studies (with reasons).

Study	Reason for Exclusion	Reference
Chang et al., 2015	No control group	[1]
Tandulwadkar et al., 2017	No control group	[2]
Zadehmodarres et al., 2017	No control group	[3]
Colombo et al., 2017	No clinical trial	[4]
Molina et al., 2018	No randomized trial	[5]
Wang et al., 2018	Inappropriate intervention arm	[6]
Coksuer et al., 2019	Retrospective study	[7]
Kim et al., 2019	No controlled arm	[8]
Chang et al., 2019	No randomized trial	[9]
Aghajanzadeh et al., 2020	No controlled arm	[10]
Maleki-Hajiagha et al., 2020	Systematic review	[11]
Frantz et al., 2020	No controlled arm	[12]
Tehraninejad et al., 2020	No randomized trial	[13]
Godha et al., 2019	Insufficient data	[14]

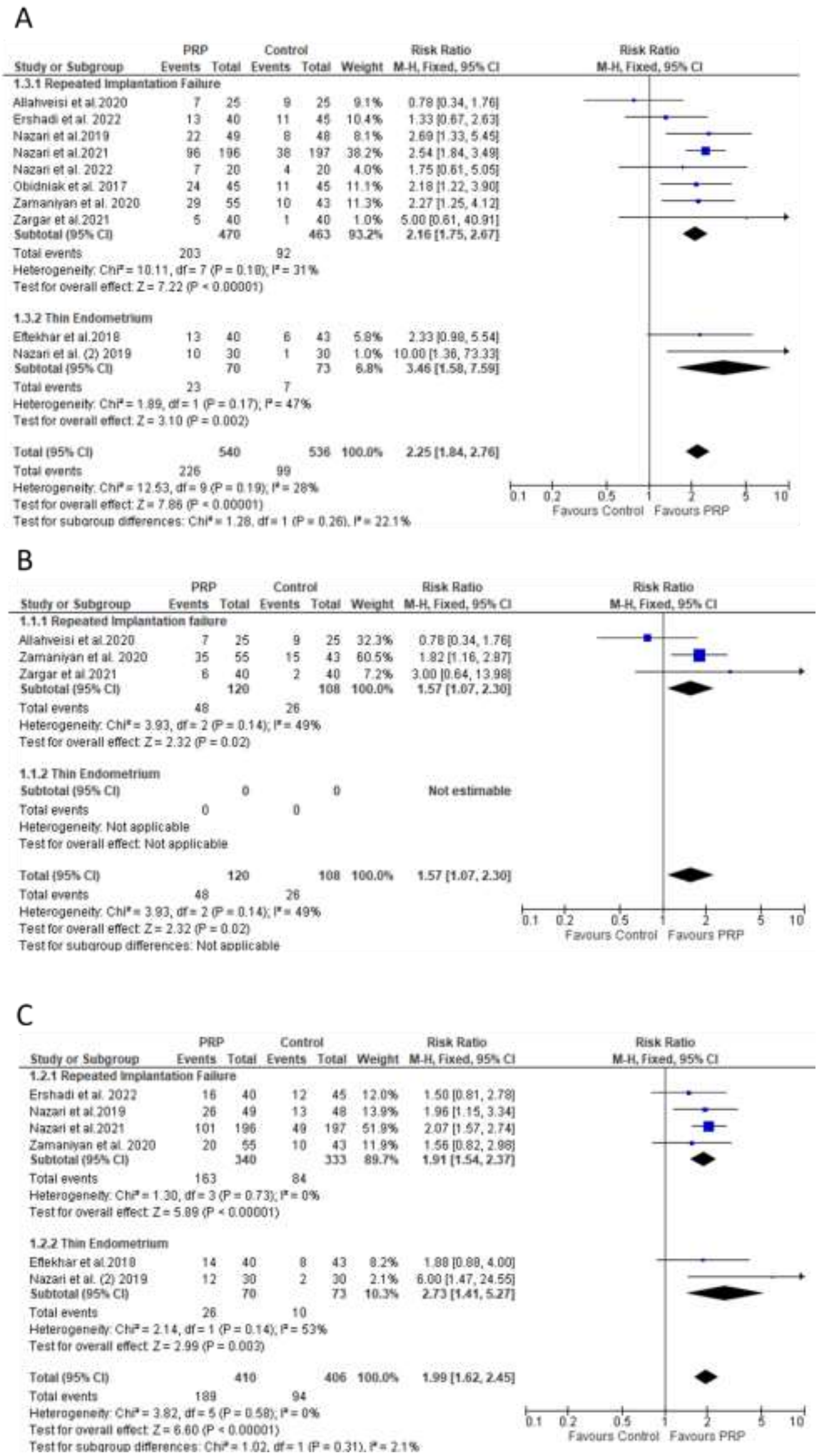
A
Study or Subgroup | PRP Events | PRP Total | Control Events | Control Total | Weight | Risk Ratio M-H, Fixed, 95% CI
1.3.1 Repeated Implantation Failure
Allahveisi et al.2020 7 25 9 25 9.1% 0.78 [0.34, 1.76]
Ershadi et al. 2022 13 40 11 45 10.4% 1.33 [0.67, 2.63]
Nazari et al.2019 22 49 8 48 8.1% 2.69 [1.33, 5.45]
Nazari et al.2021 96 196 38 197 38.2% 2.54 [1.84, 3.49]
Nazari et al. 2022 7 20 4 20 4.0% 1.75 [0.61, 5.05]
Obidniak et al. 2017 24 45 11 45 11.1% 2.18 [1.22, 3.90]
Zamaniyan et al. 2020 29 55 10 43 11.3% 2.27 [1.25, 4.12]
Zargar et al.2021 5 40 1 40 1.0% 5.00 [0.61, 40.91]
Subtotal (95% CI) 470 463 93.2% 2.16 [1.75, 2.67]
Total events 203 92
Heterogeneity: Chi² = 10.11, df = 7 (P = 0.18); I² = 31%
Test for overall effect: Z = 7.22 (P < 0.00001)
1.3.2 Thin Endometrium
Eftekhar et al.2018 13 40 6 43 5.8% 2.33 [0.98, 5.54]
Nazari et al. (2) 2019 10 30 1 30 1.0% 10.00 [1.36, 73.33]
Subtotal (95% CI) 70 73 6.8% 3.46 [1.58, 7.59]
Total events 23 7
Heterogeneity: Chi² = 1.89, df = 1 (P = 0.17); I² = 47%
Test for overall effect: Z = 3.10 (P = 0.002)
Total (95% CI) 540 536 100.0% 2.25 [1.84, 2.76]
Total events 226 99
Heterogeneity: Chi² = 12.53, df = 9 (P = 0.19); I² = 28%
Test for overall effect: Z = 7.86 (P < 0.00001)
Test for subgroup differences: Chi² = 1.28, df = 1 (P = 0.26), I² = 22.1%
0.1 0.2 0.5 1 2 5 10
Favours Control Favours PRP
B
Study or Subgroup | PRP Events | PRP Total | Control Events | Control Total | Weight | Risk Ratio M-H, Fixed, 95% CI
1.1.1 Repeated Implantation failure
Allahveisi et al.2020 7 25 9 25 32.3% 0.78 [0.34, 1.76]
Zamaniyan et al. 2020 35 55 15 43 60.5% 1.82 [1.16, 2.87]
Zargar et al.2021 6 40 2 40 7.2% 3.00 [0.64, 13.98]
Subtotal (95% CI) 120 108 100.0% 1.57 [1.07, 2.30]
Total events 48 26
Heterogeneity: Chi² = 3.93, df = 2 (P = 0.14); I² = 49%
Test for overall effect: Z = 2.32 (P = 0.02)
1.1.2 Thin Endometrium
Subtotal (95% CI) 0 0 Not estimable
Total events 0 0
Heterogeneity: Not applicable
Test for overall effect: Not applicable
Total (95% CI) 120 108 100.0% 1.57 [1.07, 2.30]
Total events 48 26
Heterogeneity: Chi² = 3.93, df = 2 (P = 0.14); I² = 49%
Test for overall effect: Z = 2.32 (P = 0.02)
Test for subgroup differences: Not applicable
0.1 0.2 0.5 1 2 5 10
Favours Control Favours PRP
C
Study or Subgroup | PRP Events | PRP Total | Control Events | Control Total | Weight | Risk Ratio M-H, Fixed, 95% CI
1.2.1 Repeated Implantation Failure
Ershadi et al. 2022 16 40 12 45 12.0% 1.50 [0.81, 2.78]
Nazari et al.2019 26 49 13 48 13.9% 1.96 [1.15, 3.34]
Nazari et al.2021 101 196 49 197 51.9% 2.07 [1.57, 2.74]
Zamaniyan et al. 2020 20 55 10 43 11.9% 1.56 [0.82, 2.98]
Subtotal (95% CI) 340 333 89.7% 1.91 [1.54, 2.37]
Total events 163 84
Heterogeneity: Chi² = 1.30, df = 3 (P = 0.73); I² = 0%
Test for overall effect: Z = 5.89 (P < 0.00001)
1.2.2 Thin Endometrium
Eftekhar et al.2018 14 40 8 43 8.2% 1.88 [0.88, 4.00]
Nazari et al. (2) 2019 12 30 2 30 2.1% 6.00 [1.47, 24.55]
Subtotal (95% CI) 70 73 10.3% 2.73 [1.41, 5.27]
Total events 26 10
Heterogeneity: Chi² = 2.14, df = 1 (P = 0.14); I² = 53%
Test for overall effect: Z = 2.99 (P = 0.003)
Total (95% CI) 410 406 100.0% 1.99 [1.62, 2.45]
Total events 189 94
Heterogeneity: Chi² = 3.82, df = 5 (P = 0.58); I² = 0%
Test for overall effect: Z = 6.60 (P < 0.00001)
Test for subgroup differences: Chi² = 1.02, df = 1 (P = 0.31), I² = 2.1%
0.1 0.2 0.5 1 2 5 10
Favours Control Favours PRP

D

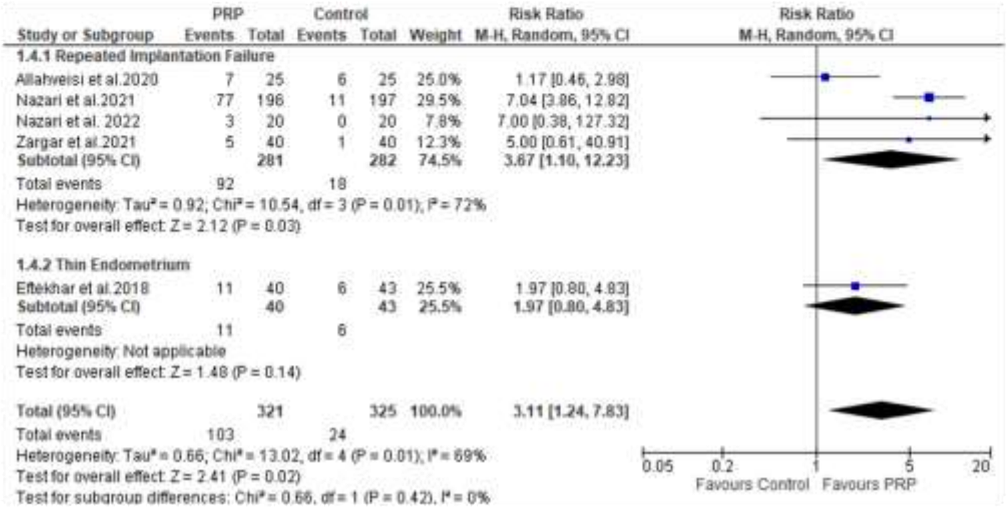

E

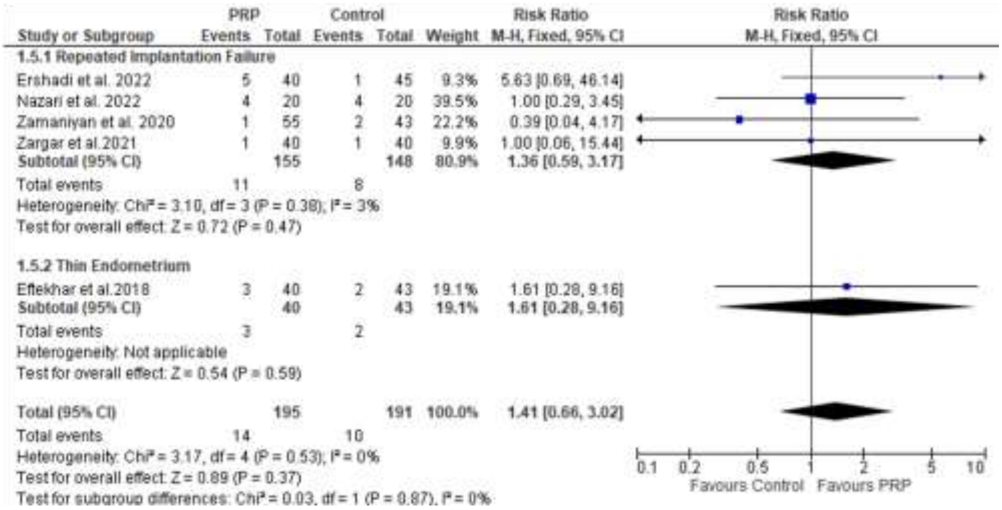

Figure S1. Meta-analysis of all studies (not excluding high-risk bias studies) evaluating (**A**) clinical pregnancy rate, (**B**) implantation rate, (**C**) biochemical pregnancy rate, (**D**) live-birth rate, and (**E**) miscarriage rate. CI: confidence interval; PRP: platelet-rich plasma.

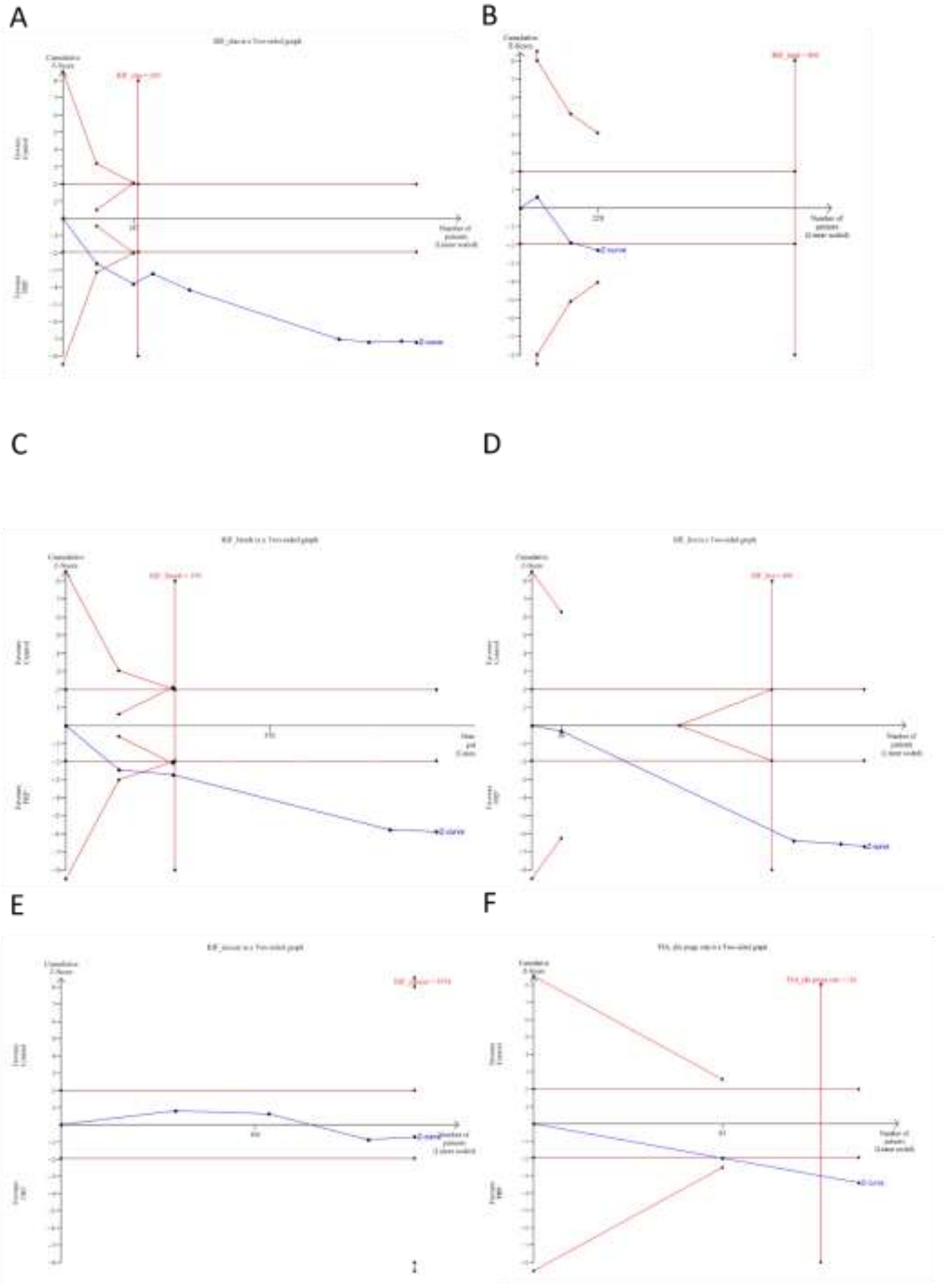

Figure S2. Trial sequential analysis for (**A**) clinical pregnancy rate, (**B**) implantation pregnancy rate, (C) biochemical pregnancy rate, (**D**) live-birth rate and (**E**) miscarriage rate in Recurrent Implantation Failure (RIF) studies. Trial sequential analysis for (**F**) clinical pregnancy in Thin Endometrium (TE) studies.

Table S3. Summary of the quality assessment by GRADE approach of outcomes included in the meta-analysis of Repeated Implantation Failure patients.

Certainty Assessment							№ of Patients		Effect			
**№ of Studies**	**Study design**	**Risk of Bias**	**Inconsistency**	**Indirectness**	**Imprecision**	**Other Considerations**	**PRP**	**Conventional Treatment**	**Relative (95% CI)**	**Absolute (95% CI)**	**Certainty**	**Importance**
**Implantation rate**												
3	randomised trials	not serious	not serious	not serious	very serious[a]	none	48/120 (40.0%)	26/108 (24.1%)	**RR 1.57** (1.07 to 2.30)	**137 more per 1000** (from 17 more to 313 more)	⊕⊕◯◯ Low	IMPORTANT
**Biochemical pregnancy rate**												
4	randomised trials	not serious	not serious	not serious	not serious	none	163/340 (47.9%)	84/333 (25.2%)	**RR 1.91** (1.54 to 2.37)	**230 more per 1000** (from 136 more to 346 more)	⊕⊕⊕⊕ High	CRITICAL
**Clinical pregnancy rate**												
8	randomized trials	not serious	not serious	not serious	not serious	strong association	203/470 (43.2%)	92/463 (19.9%)	**RR 2.16** (1.75 to 2.67)	**230 more per 1000** (from 149 more to 332 more)	⊕⊕⊕⊕ High	CRITICAL
**Live birth rate**												
4	randomized trials	not serious	serious[b]	not serious	very serious[c]	strong association	92/281 (32.7%)	18/282 (6.4%)	**RR 3.67** (1.10 to 12.23)	**170 more per 1000** (from 6 more to 717 more)	⊕⊕◯◯ Low	CRITICAL
**Miscarriage rate**												
4	randomized trials	not serious	not serious	not serious	very serious[d]	none	11/155 (7.1%)	8/148 (5.4%)	**RR 1.36** (0.59 to 3.17)	**19 more per 1000** (from 22 fewer to 117 more)	⊕⊕◯◯ Low	CRITICAL

a: Optimal information size is not met and the 95% CI of the RR included RR of 1.25; b: High heterogeneity across studies; c: Optimal information size is not met, wide range of 95%CI and the 95% CI of the RR included RR of 1.25; d: Optimal information size is not met, wide range of 95% CI and the 95% CI of the RR included RR of 0.75 and 1.25; CI: confidence interval; RR: risk ratio.

Table S4. Summary of the quality assessment by GRADE approach of outcomes included in the meta-analysis of Thin Endometrium patients.

Certainty Assessment							№ of Patients		Effect			
№ of Studies	Study Design	Risk of Bias	Inconsistency	Indirectness	Imprecision	Other Considerations	PRP	Conventional Treatment	Relative (95% CI)	Absolute (95% CI)	Certainty	Importance
**Biochemical pregnancy rate**												
2	randomized trials	Serious[a]	serious[b]	not serious	serious[c]	None	26/70 (37.1%)	10/73 (13.7%)	**RR 1.97** (1.57 to 2.48)	**133 more per 1000** (from 78 more to 203 more)	⊕◯◯◯ very low	CRITICAL
**Clinical pregnancy rate**												
2	randomized trials	serious[a]	serious[b]	not serious	serious[c]	strong association	23/70 (32.9%)	7/73 (9.6%)	**RR 3.46** (1.58 to 7.59)	**236 more per 1000** (from 56 more to 632 more)	⊕⊕◯◯ Low	CRITICAL
**Live birth rate**												
1	randomized trials	serious[a]	not serious	not serious	very serious[d]	none	11/40 (27.5%)	6/43 (14.0%)	**RR 1.97** (0.80 to 4.83)	**135 more per 1000** (from 28 fewer to 534 more)	⊕◯◯◯ very low	CRITICAL
**Miscarriage rate**												
1	randomized trials	serious[a]	not serious	not serious	very serious[e]	none	3/40 (7.5%)	2/43 (4.7%)	**RR 1.61** (0.28 to 9.16)	**28 more per 1000** (from 33 fewer to 380 more)	⊕◯◯◯ very low	CRITICAL

a: One study with unclear risk of bias; b: high heterogeneity across studies; c: optimal information size is not met; d: optimal information size is not met and the 95% of the RR included a RR value of 1.25; e: optimal information size is not met, wide range of the 95% CI of the RR and the 95% of the RR included a RR value of 0.75 and 1.25; CI: confidence interval; RR: risk ratio.

References

1. Chang, Y.; Li, J.; Chen, Y.; Wei, L.; Yang, X.; Shi, Y.; Liang, X. Autologous Platelet-Rich Plasma Promotes Endometrial Growth and Improves Pregnancy Outcome during in Vitro Fertilization. *Int. J. Clin. Exp. Med.* **2015**, *8*, 1286–1290.
2. Tandulwadkar, S.R.; Naralkar, M.V.; Surana, A.D.; Selvakarthick, M.; Kharat, A.H. Autologous Intrauterine Platelet-Rich Plasma Instillation for Suboptimal Endometrium in Frozen Embryo Transfer Cycles: A Pilot Study. *J. Hum. Reprod. Sci.* **2017**, *10*, 208–212.
3. Zadehmodarres, S.; Salehpour, S.; Saharkhiz, N.; Nazari, L. Treatment of thin endometrium with autologous platelet-rich plasma: A pilot study. *JBRA Assist. Reprod.* **2017**, *21*, 54–56.
4. Colombo, G.V.L.; Fanton, V.; Sosa, D.; Criado Scholz, E.; Lotti, J.; Aragona, S.E.; Lotti, T. Use of platelet rich plasma in human infertility. *J. Biol. Regul. Homeost. Agents* **2017**, *31*, 179–182.
5. Molina, A.; Sánchez, J.; Sánchez, W.; Vielma, V. Platelet-rich plasma as an adjuvant in the endometrial preparation of patients with refractory endometrium. *JBRA Assist. Reprod*. **2018**, *22*, 42–48.
6. Wang, X.; Liu, L.; Mou, S.; Zhao, H.; Fang, J.; Xiang, Y.; Zhao, T.; Sha, T.; Ding, J.; Hao, C. Investigation of Platelet-Rich Plasma in Increasing Proliferation and Migration of Endometrial Mesenchymal Stem Cells and Improving Pregnancy Outcome of Patients with Thin Endometrium. *J. Cell. Biochem.* **2018**, *120*, 7403–7411. https://doi.org/10.1002/jcb.28014.

7. Coksuer, H.; Akdemir, Y.; Ulas Barut, M. Improved in Vitro Fertilization Success and Pregnancy Outcome with Autologous Platelet-Rich Plasma Treatment in Unexplained Infertility Patients That Had Repeated Implantation Failure History. *Gynecol. Endocrinol.* **2019**, *35*, 815–818. https://doi.org/10.1080/09513590.2019.1597344.
8. Kim, H.; Shin, J.E.; Koo, H.S.; Kwon, H.; Choi, D.H.; Kim, J.H. Effect of Autologous Platelet-Rich Plasma Treatment on Refractory Thin Endometrium During the Frozen Embryo Transfer Cycle: A Pilot Study. *Front. Endocrinol.* **2019**, *10*, 61. https://doi.org/10.3389/fendo.2019.00061.
9. Chang, Y.; Li, J.; Wei, L.N.; Pang, J.; Chen, J.; Liang, X. Autologous platelet-rich plasma infusion improves clinical pregnancy rate in frozen embryo transfer cycles for women with thin endometrium. *Medicine* **2019**, *98*, e14062. https://doi.org/10.1097/MD.0000000000014062.
10. Aghajanzadeh, F.; Esmaeilzadeh, S.; Basirat, Z.; Mahouti, T.; Heidari, F.N.; Golsorkhtabaramiri, M. Using Autologous Intrauterine Platelet-Rich Plasma to Improve the Reproductive Outcomes of Women with Recurrent Implantation Failure. *JBRA Assist. Reprod.* **2020**, *24*, 30–33. https://doi.org/10.5935/1518-0557.20190055.
11. Maleki-Hajiagha, A.; Razavi, M.; Rouholamin, S.; Rezaeinejad, M.; Maroufizadeh, S.; Sepidarkish, M. Intrauterine Infusion of Autologous Platelet-Rich Plasma in Women Undergoing Assisted Reproduction: A Systematic Review and Meta-Analysis. *J. Reprod. Immunol.* **2020**, *137*, 103078. https://doi.org/10.1016/j.jri.2019.103078.
12. Frantz, N.; Ferreira, M.; Kulmann, M.I.; Frantz, G.; Bos-Mikich, A.; Oliveira, R. Platelet-Rich plasma as an effective alternative approach for improving endometrial receptivity—A clinical retrospective study. *J. Bras. Reprod. Assist* **2020**, *24*, 442–446. https://doi.org/10.5935/1518-0557.20200026
13. Tehraninejad, E.S.; Kashani, N.G.; Hosseini, A.; Tarafdari, A. Autologous platelet-rich plasma infusion does not improve pregnancy outcomes in frozen embryo transfer cycles in women with history of repeated implantation failure without thin endometrium. *J. Obstet. Gynaecol. Res*. **2021**, *47*, 147–151.
14. Godha, Z.; Nayar, K.D.; Gupta, S.; Singh, M.; Gupta, M.; Kant, G.; Nayar, K. Randomized controlled trial of intrauterine infusion of autologous platelet rich plasma (PRP) versus granulocyte colony stimulating factor (G-CSF) in thin endometrium in frozen embryo transfer. In *Human Reproduction*; Oxford Univ Press; Oxford, UK, 2019; Volume 34, p. 277.
